# Obituary

**Published:** 1894-08

**Authors:** 


### Obituary.

Dr. William Thompson Briggs died at his home in Nashville, June 13, 1894. Dr. Briggs was born at Bowling Green, Ky., December 4, 1828, and received his medical degree from Transylvania University in 1848. He was appointed demonstrator of anatomy in the medical department of the University of Nashville in 1851. In 1865, he was made professor of surgical anatomy and physiology; in 1866, professor of obstetrics and diseases of women and children, and in 1868, he was appointed professor of surgery, which latter chair he occupied until his death. He was one of the most distinguished surgeons of the South, and his judgment was appealed to far and wide as a consulting and operating surgeon. He was married in 1850 and his wife preceded him to the grave but a few weeks. He was a member of the American Surgical Association, of the Southern Surgical and Gynecological Association and was elected president of the American Medical Association at the Nashville meeting in 1890, presiding at the Washington meeting in 1891. His son, Dr. Charles S. Briggs, editor of the *Nashville Journal of Medicine and Surgery*, has been elected to the chair of surgery, for so many years occupied and but just vacated by his distinguished father.

